# Supplementary material for: The Challenge to Stabilize, Extract and Analyze Urinary Cell-Free DNA (ucfDNA) during Clinical Routine
Source: Diagnostics (Basel). 2023 Dec 14;13(24):3670. doi: 10.3390/diagnostics13243670 (PMC10743081; doi:10.3390/diagnostics13243670)
Supplement: Supplementary file 1 [file diagnostics-13-03670-s001.zip › diagnostics-2758739-Supplmentary Table S2.pdf]

Supplementary Table S2. Overview and analysis of samples used for ddPCR. The table displays sample numbers and corresponding information; the quantity of extracted cfDNA measured with Qubit and the respective cfDNA amount in 7 µl used per ddPCR assay; the fractional abundance (FA); the measured concentration in copies/µl and the calculated quantity of cfDNA in ng.

sample no.	Sample content	Storage conditions	DNA [ng] with Qubit (25 µl eluate)	DNA [ng] used per ddPCR assay (7µl)	no. of positive mutant droplets	FA [%]	DNA conc. [copies/µl] measured by ddPCR	Calculated** DNA [ng] per ddPCR assay [7 µl
1	NC	No buffer, 0h	6.02	1.69		-	26.60	1.93
2	Spike AF 5%	No buffer, 0h	4.16	1.16	1; 0	0	30.2	2.20
3	NC	UAS, 0h	8.89	2.49		-	47.8	3.47
4	Spike AF 5%	UAS, 0h	17.13	4.80	25; 23	2.05	85.6	6.50
5	NC	UAS, 4h RT	9.99	2.80	NA*			
6	Spike AF 5%	UAS, 4h, RT	19.33	5.41	34; 21	2.00	112.7	8.18
7	NC	UAS, 4h, F	6.99	1.96		-	48.40	3.52
8	Spike AF 5%	UAS, 4h, F	17.90	5.01	14; 14	3.20	75.95	5.51
9	NC	UAS, 24h, RT	15.40	4.31		-	88.50	6.43
10	Spike AF5%	UAS, 24h, RT	25.33	7.09	20	1.90	126.40	9.18
11	NC	UAS, 24h, F	10.40	2.91		-	58.50	4.25
12	Spike AF 5%	UAS, 24h, F	13.87	3.88	19; 30	1.80	105.20	7.64
13	NC	AlloU, 0h	2.14	0.60		-	8.05	0.58
14	Spike AF 5%	AlloU, 0h	14.47	4.05	23; 36	2.95	73.1	5.30
15	NC	AlloU, 4h, RT	1.69	0.47		-	6.20	0.45
16	Spike AF 5%	AlloU, 4h, RT	8.32	2.33	8; 3	2.25	41.70	3.03
17	NC	AlloU, 4h, F	1.87	0.52		-	7.1	0.52
18	Spike AF 5%	AlloU, 4h, F	11.97	3.35	8; 2	2.5	16.55	1.20
19	NC	AlloU, 24h, RT	3.81	1.07		-	5.35	0.39
20	Spike AF 5%	AlloU, 24h, RT	8.15	2.28	0	0	32.1	2.33
21	NC	AlloU, 24h, F	1.60	0.45		-	5.60	0.41
22	Spike AF 5%	AlloU, 24h, F	13.07	3.66	31; 18	2.00	73.60	5.34
23	NC	UCB, 0h	1.10	0.31		-	2.0	0.15
24	Spike AF 5%	UCB, 0h	3.04	0.85	3; 1	0.65	9.7	0.70
25	NC	UCB, 4h, RT	1.63	0.46		-	4.25	0.31
26	Spike AF 5%	UCB, 4h, RT	1.72	0.48	0	0	3.15	0.23

27	Ref. Standard 0.1%	***	2.8	1; 0	0	25.35	1.84
28	Ref. Standard 1%	***	2.8	3; 5	1.0	22.95	1.67
29	Ref. Standard 5%	***	2.8	34; 23	5.1	25.90	1.88

NA*: sample could not be analyzed due to insufficient droplet generation.

**Calculated as mentioned above: $\frac{ng}{assay} = \frac{copies}{\mu l} * 22\ \mu l * 0.0033\ ng$.

*** Reference standard solutions were measured fluorometrically using Qbit resulting in 400ng/ml each.
